# Reliability of Upper Limb Pin-Prick Stimulation With Electroencephalography: Evoked Potentials, Spectra and Source Localization

**DOI:** 10.3389/fnhum.2022.881291

**Published:** 2022-07-21

**Authors:** Lisa Tedesco Triccas, Kenneth P. Camilleri, Camilleri Tracey, Fahimi Hnazaee Mansoureh, Wittevrongel Benjamin, Muscat Francesca, Boccuni Leonardo, Mantini Dante, Verheyden Geert

**Affiliations:** ^1^Department of Rehabilitation Sciences, KU Leuven, Leuven, Belgium; ^2^Department of Systems and Control Engineering, University of Malta, Msida, Malta; ^3^REVAL Rehabilitation Research Center, Faculty of Rehabilitation Sciences, Hasselt University, Hasselt, Belgium; ^4^Centre for Biomedical Cybernetics, University of Malta, Msida, Malta; ^5^Laboratory for Neuro- and Psychophysiology, KU Leuven, Leuven, Belgium; ^6^The Wellcome Trust Centre for Neuroimaging, University College London Institute of Neurology, London, United Kingdom; ^7^Institut Guttmann, Institut Universitari de Neurorehabilitació Adscrit a la Universitat Autónoma de Barcelona, Barcelona, Spain; ^8^Universitat Autònoma de Barcelona, Cerdanyola del Vallès, Spain; ^9^Fundació Institut d’Investigació en Ciències de la Salut Germans Trias i Pujol, Barcelona, Spain; ^10^Department of Movement Sciences, KU Leuven, Leuven, Belgium

**Keywords:** psychometric, exteroception, hand, cortical activity, event-related potentials, frequency band, sources

## Abstract

In order for electroencephalography (EEG) with sensory stimuli measures to be used in research and neurological clinical practice, demonstration of reliability is needed. However, this is rarely examined. Here we studied the test-retest reliability of the EEG latency and amplitude of evoked potentials and spectra as well as identifying the sources during pin-prick stimulation. We recorded EEG in 23 healthy older adults who underwent a protocol of pin-prick stimulation on the dominant and non-dominant hand. EEG was recorded in a second session with rest intervals of 1 week. For EEG electrodes Fz, Cz, and Pz peak amplitude, latency and frequency spectra for pin-prick evoked potentials was determined and test-retest reliability was assessed. Substantial reliability ICC scores (0.76–0.79) were identified for evoked potential negative-positive amplitude from the left hand at C4 channel and positive peak latency when stimulating the right hand at Cz channel. Frequency spectra showed consistent increase of low-frequency band activity (< 5 Hz) and also in theta and alpha bands in first 0.25 s. Almost perfect reliability scores were found for activity at both low-frequency and theta bands (ICC scores: 0.81–0.98). Sources were identified in the primary somatosensory and motor cortices in relation to the positive peak using s-LORETA analysis. Measuring the frequency response from the pin-prick evoked potentials may allow the reliable assessment of central somatosensory impairment in the clinical setting.

## Introduction

After an unexpected stimulus such as a pin-prick, the human brain integrates that input in the somatosensory system. Specifically, the primary and secondary somatosensory cortex, the thalamus, the insula, the posterior parietal cortex and the cerebellum all play important parts in processing somatosensory information (Abraira and Ginty, 2013; Limanowski et al., 2020). Understanding the changes in cortical mechanisms involved in somatosensory processing in neurological conditions could be the next step in predicting recovery and identifying different treatment programs for patients.

Routine measurement involving behavioral assessments in neurological conditions such as stroke could predict the functional outcome of the upper limb (Boccuni et al., 2018; Zandvliet et al., 2020). However, they mostly lead to dichotomous results and are sometimes unable to detect change. EEG is an accessible, safe and non-invasive method that measures changes in brain activation from sensory stimuli (Shiner et al., 2015; Van Diessen et al., 2015). EEG also has almost no contra-indications, which makes it a safe application in healthy adults or any clinical population (Borich et al., 2015b). Identifying high reliability of sensory modalities in combination with EEG could be a promising tool for prediction models of recovery from a brain injury (Triccas et al., 2019). This study aimed to evaluate the reliability of the combination of measures to be further used in people with stroke with upper limb somatosensory impairments.

A somatosensory modality that could be combined with EEG is exteroception, induced by electrical nerve stimulation, heat, laser, vibration or pin-prick. Mechanical pin-prick stimulates the type I A-fiber mechano-heat (I-AMH) nociceptors and has been combined with EEG in a few studies involving healthy young adults (Iannetti et al., 2013; van den Broeke et al., 2015, 2016). From pin-prick stimulation, oscillatory activity at different frequency bands and evoked potentials, which are linked to short and immediate neuronal response, can also be sensitively measured and analyzed. Study of change in frequency band activity gives information about the functional state of the sensorimotor cortex (Illman et al., 2020). The advantage of using pin-prick stimulation over median nerve electrical stimulation as a tactile stimulus, is that the latter targets both muscle efferent and joint afferent fibers resulting muscle contraction could be elicited upon stimulation disturbing the sensory response (Fujii et al., 1994; Kandel et al., 2000; Mauguiere, 2005). In stroke rehabilitation, it is sometimes necessary to separate the somatosensory analysis from the motor one for example exploring the effect of a somatosensory rehabilitation program on upper limb function.

Pin-prick evoked potentials (PPEP) have been shown to consist of a biphasic negative-positive (N-P) wave with average peak latencies of 111 and 245 ms and average amplitudes of 3.5 and 11.1 μV in young healthy adults (Iannetti et al., 2013). Recently, Rosner et al. (2020) explored the reliability of PPEP measurement. They identified a poor reliability (ICC = 0.27) of N-P amplitude measurement at dermatome C6 but higher reliability of N2-latency (ICC = 0.63) in healthy younger and older adults. The poor to moderate reliability values could be due to researchers administering only 15 pin-prick stimuli. A higher number of stimuli of >500 is recommended for recording somatosensory-evoked potentials (Cruccu et al., 2008). van den Broeke et al. (2017) also explored a time-frequency analysis from PPEPs resulting in a phase-locked low frequency response, followed by a drop in the alpha-band oscillations. They concluded that time-frequency analysis is more sensitive than time-domain analysis. However, they did not examine the reliability of this assessment using time-frequency analysis (Asadzadeh et al., 2020).

Identifying the sources in the brain through source localization analysis from N-P response from pin-prick stimuli though also warranted, has never been conducted to our knowledge. EEG source localization is potentially capable of identifying sources that are not captured by functional Magnetic Resonance Imaging (fMRI). EEG has a high temporal resolution and unlike fMRI, it is also a direct reflection of neural activity (Cohen, 2017; Michel and He, 2019). In comparison, laser evoked potentials, which stimulate type II Aδ mechano-heat receptors, have also resulted in an initial negative–positive complex in which sources have been contradictory in either the parasylvian region or the contralateral S1 contribution using both dipole and source estimation analysis (LORETA) (Treede et al., 2000; Valentini et al., 2012). Similarly, EEG dipole analysis after median nerve stimulation also resulted in early sources (at P50) located in the contralateral S1, medial frontal gyrus, the insula, and also hippocampus (Bak et al., 2011).

Therefore, the novelty of this research was to assess the variability of EEG in combination with pin-prick assessment on separate days (test-retest reliability) in two different populations using time-domain and time-frequency analyses. Based on similar research using a similar somatosensory modality, we hypothesized that substantial reliability will be identified in the time-domain (Özgül et al., 2017) and time-frequency analysis. Additionally, to estimate the sources and their variability in the brain from the N-P complex from pin-prick stimulation using standardized low-resolution brain electromagnetic tomography source analysis (sLORETA) (Pascual-Marqui, 2002). We hypothesized that similar sources to laser evoked potentials in the contralateral S1 (Valentini et al., 2012) will be identified from pin-prick stimulation in sessions 1 and 2. The research involved healthy adults above the age of 40 to be used as age-and-gender matched to patients with stroke, presented in a separate study.

## Materials and Methods

### Participants

Twenty-three healthy adult participants from two different centers, Malta and Belgium, participated in this study. Each subject participated in two sessions of EEG with pin-prick stimulation in 1 week. All participants were provided an information pack explaining the aim of the study and they all gave informed consent according to the declaration of Helsinki. The participants had no previous peripheral nerve injury in the upper limbs and were screened by the reliable Erasmus Modified Nottingham Sensory Assessment (EmNSA) (Stolk-Hornsveld et al., 2006). The EmNSA assesses tactile sensation and proprioception in the extremities. The participants also confirmed that they were not diagnosed with diabetes and other neurological conditions. Level of cognitive function was screened by the Montreal Cognitive Assessment (MOCA) (Nasreddine et al., 2005) in Belgium or the Mini-Mental State Examination (MMSE) (Folstein et al., 1983) in Malta. Approval was obtained from the University of Malta Research Ethics Committee (Registration number: 002/2016) and the UZ/KU Leuven Ethics Committee (Registration number: S61174).

### Experimental Setup

EEG data was collected by four researchers at the Experimental Neurology Department of Gasthuisberg University Hospital Leuven, in Belgium and at the Biomedical Engineering Laboratory, Faculty of Engineering, University of Malta. The EEG protocol consisted of placing electrodes on the head according to the extended 10–20 international system (Homan et al., 1987). During EEG recording, participants sat in a chair as still as possible with their gaze fixed and their hand palms facing downwards. A BioSemi (Netherlands) EEG system, consisting of 64 active shielded Ag-AgCl electrodes mounted in an elastic electrode-cap was used in Belgium. FCz channel was registered as the reference and AFz channel as the ground. A g. tec (Austria) EEG system with 32 g. Scarabeo sintered Ag-AgCl ring electrodes also mounted in a cap was used in Malta. For this system, the AFz channel was registered as the ground and the reference clip was attached to the left ear lobe of the participant. Electrodes were concentrated on the parietal, central and frontal regions. The electrode cap was placed so that Cz electrode was exactly in the middle of an imaginary line drawn from the occipital tuberculum posterior to the middle of the nasal bridge. Underlying hair and dead skin cells were minimized by using a cotton swab and subsequently conductive gel to ensure good contact between the electrodes and the scalp.

### Data Acquisition

**FIGURE 1:**
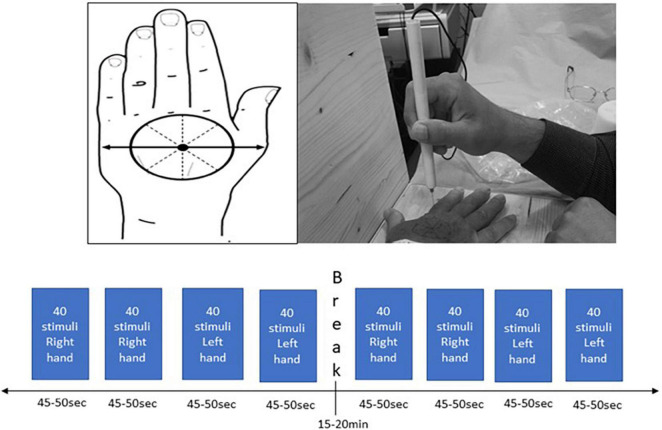
Standardized hand marking for the pin-prick stimulation protocol on the dorsum of both hands.

In order to administer a sharp stimulus, a pinprick stimulator (MRC Systems, Germany) was used, which was interfaced with the EEG systems. The left or right hand was placed on a wooden apparatus that ensured that participants were not allowed to see when pin-prick stimulation was applied. In order to apply the stimuli to each hand in a standardized manner at dermatomes C6/C7, the distance between the base of metacarpal I to the middle of metacarpal V was measured. The middle of this distance was set as the center of the circle that was drawn on the dorsum of the hand. This circle was divided in eight equal compartments and was drawn on both hands (Figure 1). Ten sets of 8 stimuli (one stimulus per compartment) with approximately 6 s apart were applied in a random order to the dorsum of both hands aiming to apply 160 stimuli per hand. To ensure that the same force of pin-prick stimulation was applied to the participants, a training program with all researchers was conducted. During the experiment, participants had to rate their level of sensation from 0 to 100% which was recorded on a mobile phone.

### Data Processing and Analyses

The mean and standard deviation of level of sensation scores and number of epochs per session for both hands was calculated in SPSS. Wilcoxon signed rank test was used to assess the differences in sensation scores between sessions setting a significance level at *p* < 0.05. EEG data was processed off-line in Matlab (Mathworks^®^ version R2018a Update 6, Natick, Maine, United States) using a combination of scripts from EEGLAB (Swartz Centre for Computational Neuro-science, eeglab 14_1_2b) (Brunner et al., 2013). The EEG signal of patients was digitized at 2,000 Hz in Belgium and at 256 Hz in Malta. All data was then down sampled to 256 Hz and then band pass-filtered between 1 and 50 Hz, to remove line-noise and slow drifts, and to improve the frequency specificity of subsequent post-processing techniques. This was followed by average referencing (Bertrand et al., 1985).

For time-domain analysis, EEG epochs that included the pin-prick stimuli were extracted with a window of –0.5–1.5 s after stimulation. EEG epochs were averaged across trials, separately for each hand. Filtered epochs were then baseline-corrected with a reference interval of –0.5–0 s. Epochs contaminated by artifacts due to eye blinks or movements were corrected with an independent component analysis (ICA) algorithm (Comon, 1994). The elicited PPEPs were first visually assessed in EEGLAB (Lee et al., 2013). A script was used to calculate the amplitudes (max/min) and corresponding latencies of different peaks of the PPEPs on channels Cz, C3, C4, Fz and Pz channels.

Time-frequency analysis involved two stages. First, time-frequency representations (TFRs) were calculated in Brainstorm (Tadel et al., 2011) within the frequency range of 3–40 Hz for a time window from –500 to 1,500 ms for each subject using the Morlet wavelet transformation for the right and left hands, sessions 1 and 2 (Tallon-Baudry et al., 1997; Illman et al., 2020). This was followed by spectrum (1/f compensation) and baseline normalization (–500–0.5 ms), choosing the method Event-related perturbation: ERS/ERD. From the ERS/ERD plots, frequency band activity was observed. Further time-frequency analysis was then carried out in Matlab to explore spontaneous frequency band activity (Illman et al., 2020). Specifically, the power spectral density (psd) was computed using short-time Fourier transform and then the peaks within the 0–1 s window, for the 0–5 Hz, 6–10 Hz (van den Broeke et al., 2017), 8–12.5 and 13–30 frequency bands, were identified for both sessions. The time at which these peaks occurred, as well as the relative amplitudes, were also recorded. The relative amplitude is defined as the amplitude of the peak relative to the highest peak within the analysis time window, giving a measure of the significance of the detected peak with the 0 –1 s window.

Source imaging analysis for all participants was performed using Brainstorm (Tadel et al., 2011). The standard ICBM152 anatomy included in Brainstorm was used to construct the forward solution using a realistic three-layer head model (OpenMEEG BEM) where the source space was constrained to a template MRI volume with 15,000 vertices and relative conductivities of 1, 0.0125 and 1 for the scalp, skull, and brain layers, respectively (Gramfort et al., 2010; Hnazaee et al., 2020). Source localization of EEG data recorded at the scalp can be a challenging problem. Different algorithms using alternative approaches have been developed to estimate the location of EEG sources (Grech et al., 2008). In our analysis, we used the standardized LORETA (sLORETA) algorithm (Pascual-Marqui, 2002), which has been identified as an accurate method of identifying sources and used in similar studies exploring somatosensory stimulation in combination with EEG (Babiloni et al., 2007; Ertl et al., 2020). The noise covariance matrix required as input for source localization with sLORETA was obtained by merging the matrices calculated from the baseline of the averaged trials. In order to visualize in time the averaged PPEPs, contact sheets for right and left stimulation as intraindividual analysis set at an amplitude of 70% for the right and 60% for the left were generated. Regions of interest were identified using Desikan-Killiany (Desikan et al., 2006) and Brodmann areas (Kaas et al., 1979) and peak activity was visualized using scout time series analysis for session 1 and session 2 for both hands.

Combining data from Belgium and Malta, the test-retest reliability of peak power amplitudes and latencies and peak frequency latencies at ranges 0–5, 6–10, 8–12.5, and 13–30 Hz, for matched trials of relative amplitude 0.4 and above of the PPEPs for both sessions were analyzed by Intraclass Correlation Coefficient (ICC). The latter involved using single measures in a two-way mixed model (Model 3, 1) followed by Bland and Altman Analysis in IBM SPSS Statistic 25. ICC was calculated by mean squares (i.e., estimates of the population variances based on the variability among a given set of measures) obtained through analysis of variance. ICC scores were evaluated on the following agreement level: 0.2–0.4 fair, 0.4–0.6 moderate, 0.6–0.8 substantial, and > 0.8 almost perfect (Landis and Koch, 1977). Bland and Altman figures were plotted for ICC scores > 0.75 to allow for a visual interpretation of measurement agreement focusing on a reference range within which 95% of all differences between measurements could lie (Bland and Altman, 1986; Mansournia et al., 2021).

## Results

Overall the data quality was good for all participants. The mean epochs for the right hand session 1 were 139 (*SD*: 31) and session 2 were 143 (*SD*: 28). The mean epochs for the left hand session 1 were 149 (*SD*: 23) and session 2 were 150 (*SD*: 23). Demographics of the participants constituted of: mean (SD) age of the participants was 62.48 (± 11.47) years, 13 were females and 10 were males and 21 were right-handed and 2 were left-handed. The mean (SD) MOCA score for participants in Belgium was 26.38 (3.57) and mean (SD) MMSE for participants in Malta was 29.44 (0.83). The mean pain rating scores for the right hand at sessions 1 and 2 were 34.83 (*SD*: 24.05) and 31.29 (*SD*: 25.41) and for the left hand 35.96 (*SD*: 21.21) and 30.77 (*SD*: 23.83), respectively. The differences in the mean pain rating scores between sessions were non-significant (*p* = 0.051 right; *p* = 0.333 left).

### Time-Domain Analysis

**FIGURE 2:**
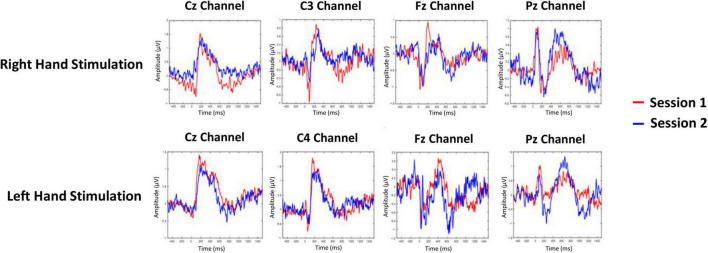
Grand-averaged pin-prick evoked potential response at Cz, C3/C4, Fz, and Pz channels for right and left hand. Response in color red is depicted for session 1 and response in color blue is depicted for session 2.

The averaged PPEP for all participants comprised of N-P complex followed by a smaller second peak (Figure 2). For the right hand, N-P mean amplitude for Cz, C3, and Fz channels ranged from 2.66 to 2.89 μV, with N-Latency ranged from 68.95 to 80.46 ms and P-Latency 147.54–176.55 ms (Table 1).

**TABLE 1:** The mean N-P amplitude and latency with respective intraclass correlation coefficient values of the first peak of the PPEP at the Cz, C3/C4, Fz, and Pz channels.

	Right hand						Left hand					
	
Channel name	N-P amplitude		N-Latency		P-Latency		N-P amplitude		N-Latency		P-Latency	
						
	Mean (*SD*) (μV)	ICC (95% CI)	Mean (*SD*) (ms)	ICC (95% CI)	Mean (*SD*) (ms)	ICC (95% CI)	Mean (*SD*) (μV)	ICC (95% CI)	Mean (*SD*) (ms)	ICC (95% CI)	Mean (*SD*) (ms)	ICC (95% CI)
Cz	2.89 (2.14)	0.573 (0.151, 0.897)	68.95 (38.80)	0.723 (0.110, 0.938)	147.54 (74.13)	0.793 (0.539, 0.915)	2.95 (1.36)	0.624 (0.262, 0.832)	63.43 (40.16)	0.487 (0.069, 0.769)	196.28 (64.60)	0.436 (0.038, 0.714)
C3/C4	2.66 (1.23)	0.763 (0.494, 0.899)	74.84 (38.85)	–0.122 (–0.527, 0.328)	171.77 (73.81)	0.428 (0.208, 0.709)	2.77 (1.44)	0.601 (0.228, 0.821)	51.50 (39.20)	0.159 (–0.294, 0.554)	169.04 (77.01)	0.272 (0.182, 0.631)
Fz	2.71 (1.90)	0.312 (–0.106, 0.636)	80.46 (73.76)	–0.114 (–0.485, 0.305)	176.55 (98.61)	0.334 (–0.082, 0.650)	2.34 (1.76)	0.537 (0.160, 0.778)	93.60 (74.06)	0.327 (–0.100, 0.652)	157.06 (71.79)	0.265 (–0.166, 0.611)
Pz	2.69 μV (1.47)	0.198 (–0.284, 0.599)	186.42 (66.61),	0.141 (–0.336, 0.561)	122.05 (88.86)	0.397 (–0.019, 0.696)	2.09 (1.36)	0.489 (0.027, 0.779)	148.82 (76.94)	0.087 (–0.397, 0.534)	119.10 (87.04)	0.482 (0.086, 0.747)

For the left hand, NP mean amplitude for Cz, C4 and Fz ranged from 2.09 to 2.95 μV, N-Latency ranged from 51.50 to 93.60 ms and the P-Latency ranged from 157.06 to 196.28 ms (Table 1). For both hands, a negative peak was seen after a positive peak at Pz channel. The N-P or P-N complex was followed by a second late positive smaller peak [1.04 μV, 445.89 ms (C3 channel); 1.55 μV, 449.52 ms (Cz channel); 1.35 μV, 452.18 ms (Fz channel); 1.22 μV, 501.66 ms (Pz channel) right hand; 1.04 μV, 445.89 ms (C3 channel); 1.55 μV, 449.52 ms (Cz channel); 1.35 μV, 452.18 ms (Fz channel); 1.22 μV, 501.66 ms (Pz channel) left hand].

### Time-Frequency Analysis

**FIGURE 3:**
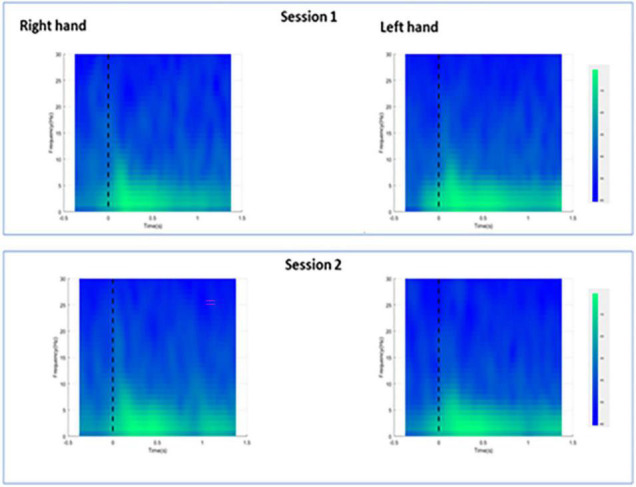
Averaged spectra for all participants during right and left hand pin-prick stimulation at the Cz channel.

**FIGURE 4:**
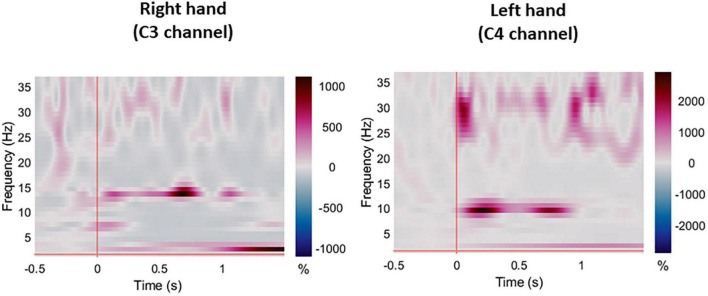
Averaged ERS/ERD plots for all participants during right and left hand pin-prick stimulation at the respective C3 and C4 channels.

For session 1, left and right pinprick stimulus elicited an early marked increase of low-frequency activities (< 5 Hz) (Figure 3) extending at mean peak latency of 0.262 s (*SD*: 0.159) at the right hand and 0.251 s (*SD*: 0.169) at the left hand at the Cz channel. Changes in the 6–10 Hz occurred earlier at 0.160 ms (*SD*: 0.070) at the right hand and 0.146 (0.076) at the left hand. Similar results were found at C3 or C4 channel (Table 2). From ERS/ERS analysis, activity in the alpha (8–3 Hz) and beta bands (13–30 Hz) were also visualized extending at mean peak latencies from 0.060 to 0.160 s (Table 2 and Figure 4). However, the latter analysis was not consistent as seen in the low-frequency analysis.

**TABLE 2:** Showing the mean (SD) peak times at 0–5, 6–10, 8–12.5, and 13–30 Hz and respective intraclass correlation coefficients (95% CI) at both hands at Cz channel and C3 channels for right hand stimulation and C4 for left hand stimulation.

Right hand									Left hand							
	
Channel name	Mean (*SD*) Actual peak time (s) 0–5 Hz	ICC (95% CI)	Mean (*SD*) Actual peak time (s) 6–10 Hz	ICC (95% CI)	Mean (*SD*) Actual peak time (s) 8–12.5 Hz	ICC (95% CI)	Mean (*SD*) Actual peak time (s) 13–30 Hz	ICC (95% CI)	Mean (*SD*) Actual peak time (s) 0–5 Hz	ICC (95% CI)	Mean (*SD*) Actual peak time (s) 6–10 Hz	ICC (95% CI)	Mean (*SD*) Actual peak time (s) 8–12.5 Hz	ICC (95% CI)	Mean (SD) Actual peak time (s) 13–30 Hz	ICC (95% CI)
Cz	0.222 (0.103)	0.977 (0.947, 0.990)	0.160 (0.070)	0.902 (0.783, 0.957)	0.165 (0.832)	0.869 (0.693, 0.848)	0.075 (0.488)	0.813 (0.447, 0.946)	0.251 (0.169)	0.978 (0.945, 0.991)	0.146 (0.076)	0.874 (0.726, 0.945)	0.141 (0.064)	0.601 (0.239, 0.816)	0.078 (0.050)	0.663 (0.226, 0.878)
C3/C4	0.238 (0.111)	0.926 (0.826, 0.969)	0.116 (0.048)	0.824 (0.629, 0.921)	0.106 (0.676)	0.585 (0.204, 0.812)	0.064 (0.553)	0.666 (0.108, 0.905)	0.189 (0.076)	0.866 (0.705, 0.942)	0.124 (0.074)	0.805 (0.517, 0.918)	0.099 (0.060)	0.751 (0.421, 0.905)	0.105 (0.089)	0.861 (0.648, 0.949)

### Source Estimation Analysis

**FIGURE 5:**
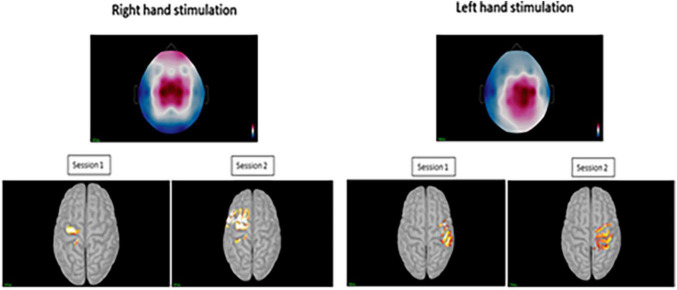
Top images displaying the topography at sensor level during the positive peak activity of the pin-prick evoked potential for the right and left hand. Bottom figures displaying the averaged peak activity at the precentral and postcentral gyri for sessions 1 and 2 at both hands.

For the displaying what is happening in time during P-Peak, sLORETA identified similar peak activity at the contralateral post-central gyrus (Brodmann areas 3b and 1) especially for right hand stimulation, indicating contralateral S1 sources. There seemed to be increased activity in the superior frontal area for the right hand stimulation for session 2 however, this was not consistent for the other sessions. Additional pre-central gyri activation was noted in left hand stimulation (Figure 5). No distinctive activity was identified during the N-peak of the PPEP.

### Reliability Analysis

**FIGURE 6:**
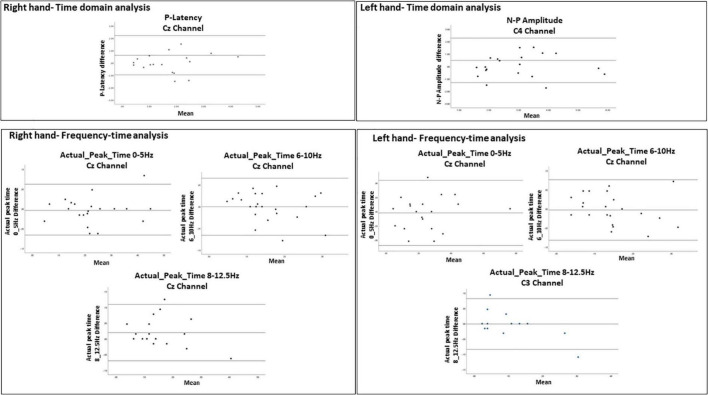
Bland-Altman plots demonstrating the 95% limits of agreement between 1st and 2nd sessions for variables with ICC = 0.75. The middle line represents the mean. The upper and lower line represent the upper and lower limits of agreement (mean ± 1.96 *SD*), respectively. The Bland-Altman analysis could not be performed for actual_peak_time analysis 0_5 Hz for C3 channel because the difference was found significant (*p* = 0.017).

For the evoked potentials, at Cz and C4 channels, substantial reliability for P-Latency [ICC: 0.793 (0.539, 0.915) right hand] and N-P amplitude [ICC: 0.763 (0.494, 0.899) left hand] respectively, were found (Table 1 and Figure 6). The results for frequency analysis are focused on the electrodes in the central region (Cz/C3/C4) since substantial reliability were identified in these regions. Almost perfect reliability values were found for time-frequency analysis between peak times at 0–5 and 6–10 Hz at sessions 1 and 2 showing ICC scores ranging from 0.977 to 0.824 for the right hand and 0.978–0.805 for the left hand (Table 2 and Figure 6). Substantial reliability values were also found for 8–12.5 and 13–30 Hz analysis (ICC: 0.813–0.869) at Cz channel for the right hand. For the left hand, higher reliability values were found at the C4 channel compared to the Cz channel for the latter analysis (ICC: 0.751–0.861).

## Discussion

To our knowledge, this is the first study exploring the sources of the N-P complex from pin-prick stimulation. Reliable sources were identified in the contralateral post and pre-central gyri especially for right hand stimulation indicating primary somatosensory and motor cortices activation. The presented research was also novel to explore the reliability of time-frequency analysis from pin-prick stimulation providing insight into the functional state of the sensorimotor cortex. Almost perfect reliability was identified for this type of analysis. Therefore, we recommend that both frequency and source analysis using sLORETA could be valuable source of exploring somatosensory impairments in neurological conditions. However, inconsistent reliability values were identified for latency and amplitude of the PPEPs at the left and right hand from the time-domain analysis. Using the latter measure to evaluate efficacy of upper limb sensorimotor rehabilitation programs cannot be considered.

From the time-domain analysis, the latency of the evoked potentials of this study matched well with similar studies (Iannetti et al., 2013; van den Broeke et al., 2015, 2016), although the identified peak amplitudes were relatively smaller. This difference could be explained by the age of participants since our population was older compared to the young participants between 23 and 30 years in similar research. Further investigation of the impact of age on evoked potential amplitude is warranted. Implementing a reliable neurophysiological assessment for assessing somatosensory impairments is important for future stroke research, since exteroceptive impairments can be present in 41–50% of patients with acute stroke (Meyer et al., 2016). We identified substantial reliability for P-Latency at the right hand and N-P amplitude for the left hand but moderate reliability for P-Latency for the left hand and N-P amplitude for the right hand. This result was not expected. One possible reason for this is the thickness of pin-prick filament. Recently, Rosner et al. (2020), identified that 256 mN stimulation intensity led to higher reliable values than the 128 mN which was used in our study (Rosner et al., 2020). The main reason for choosing a smaller force was to minimize pain through the lateral spinothalamic tract but also to explore the response to crude touch and pressure from pin-prick stimulation through the anterior spinothalamic tract. Incidentally, pin-prick stimulation could be painful however, from our experiment participants reported pain levels with mean scores of 30%, which is low.

As presented in similar research, pin-prick stimulation resulted in a low frequency response (< 5 Hz) in both sessions (van den Broeke et al., 2017). As identified in a recent magnetoencephalography (MEG) study, stimuli which were repeated and expectations that were established, resulted in associated activity in the theta and beta bands (Andersen and Lundqvist, 2019). We also found reliable peak activity in the alpha band between 6–10 and 8–13 Hz but we identified this change between 0.146 and 0.160 ms which is earlier compared to previous research (van den Broeke et al., 2017). A reduction in alpha band has been linked with somatosensory information processing (Pfurtscheller et al., 1996; Jensen and Mazaheri, 2010). In similar studies, EEG and MEG assessment with other sensory modalities such as proprioception or vibrotactile stimuli also resulted in rhythmic oscillatory activity at the alpha (8–12 Hz) and also the beta band (13–30 Hz) (Kim et al., 2020). Our ERS/ERD plots also showed activity at the beta band which had substantial reliability between sessions. During tactile stimuli, suppression of beta band has been identified in the contralateral hemisphere using MEG (Gaetz and Cheyne, 2006). A 20 Hz rhythm has also been modulated from proprioception and tactile stimulation in people with stroke; However, the reliability of the latter assessment has never been explored (Illman et al., 2020). The presence or absence of alpha and beta band frequency response in people with pin-prick impairment could give an indication of the level of recovery of upper limb somatosensory and even motor impairments in the acute stage of stroke.

As for previous research involving laser-evoked potentials and median nerve stimulation (Bak et al., 2011; Valentini et al., 2012), we can accept our hypothesis that pin-prick stimulation results in sources within the contralateral S1. This was more pronounced during right hand stimulation. In addition, sources within the contralateral M1 were also identified which is similar to what was identified in fMRI data involving median nerve stimulation (Spiegel et al., 1999). This is as expected since S1 is the primary area for sensorimotor integration (Borich et al., 2015a). For the negative peak response, inconsistent sources were identified. The negative peaks found in our participants were and earlier compared those found in laser evoked potentials which relate to activity in this area as a response to pain (Strigo et al., 2005). Compared to median nerve stimulation, negative responses have been shown to be generated within the temporal lobe at 72–96 ms (Tesche, 2000). This could be referred to as source location of SII in the upper bank of the Sylvian fissure, i.e., temporal region (Vogel et al., 2003). From MEG and fMRI studies, it was shown that tactile topographic representation was identified in the supra-sylvian cortex [including the secondary somatosensory area (S2)], indicating that this area may contribute to touch localization (Maeda et al., 1999; Del Gratta et al., 2002). However, we used a small amount of channels compared to previous research using MEG which could have contributed to the lack of consistency in identifying sources.

Apart from identifying novel findings, some limitations from this research need to be recognized. The experiments were conducted in two centers with two different EEG systems and raters. The preliminary data was always checked before data collection on the present sample and also training was provided for each rater; However, the environmental differences could have impacted the level of reliability results. In order to obtain sufficient data, the experimental procedure was quite long which lasted around 45 min in addition to the EEG setup. At the end of the second session, participants were often tired and sometimes disinterested in the experiment. Averaged referencing could have also contributed to the variability in the PPEP responses between sessions (Yao et al., 2019). Since we did not have a MRI of the healthy participants, source localization was conducted on a standard MRI implemented in Brainstorm. Therefore, identifying the exact coordinates in relation to the cortical activation might have not been identical. We used 32 electrodes in 10 of the recruited participants. Mislocalizations could occur with a low number of channels (Srinivasan et al., 1998). However, well-defined focal activity was expected from pin-prick stimulation and therefore, having 32 electrodes could give valuable insight of the underlying sources (Michel and Brunet, 2019).

## Conclusion

The main aim of this research was to explore the variability of pin-prick measurement with EEG. Almost perfect reliability was identified for response at low frequency and theta bands from upper limb pin-prick stimulation was identified. Additionally, measurement of evoked potential latency of the positive peak has also substantial reliability. As hypothesized, the positive peak resulted in sources from the primary somatosensory cortex. We can conclude that pin-prick stimulation could be used in the clinical population to assess somatosensory impairments, however, focusing on the time-frequency could be a more reliable method than the time-domain analysis.

## Data Availability Statement

The raw data supporting the conclusions of this article will be made available by the authors, without undue reservation.

## Ethics Statement

All study procedures were approved by the University of Malta Research Ethics Committee (Registration number: 002/2016) and the UZ/KU Leuven Ethics Committee (Registration number: S61174). The patients/participants provided their written informed consent to participate in this study.

## Author Contributions

LTT: development of protocol, data collection and analyses, and writing of the manuscript. KC, CT, MD, and VG: development of protocol, data analyses, and review of the manuscript. FM: data analyses and review of the manuscript. WB: data analysis. BL and MF: data collection. All authors contributed to the article and approved the submitted version.

## Conflict of Interest

The authors declare that the research was conducted in the absence of any commercial or financial relationships that could be construed as a potential conflict of interest.

## Publisher’s Note

All claims expressed in this article are solely those of the authors and do not necessarily represent those of their affiliated organizations, or those of the publisher, the editors and the reviewers. Any product that may be evaluated in this article, or claim that may be made by its manufacturer, is not guaranteed or endorsed by the publisher.
